# Co-Filtered Versus Carbon-Paste-Bonded *p*–*n* Junctions in Cold-Source-Free Single-Walled Carbon Nanotube Thermoelectric Generators

**DOI:** 10.3390/ma19194148

**Published:** 2026-09-29

**Authors:** Hiroto Nakayama, Ryota Tamai, Yuta Asano, Masayuki Takashiri

**Affiliations:** Research Institute of Science and Technology, Tokai University, Hiratsuka 259-1292, Japan; 5cajm035@tokai.ac.jp (H.N.); rt20152976@gmail.com (R.T.); 6cajm003@tokai.ac.jp (Y.A.)

**Keywords:** single-walled carbon nanotubes, *p*–*n* junction, co-filtered junction, carbon-paste bonding, thermoelectric generators, maximum power output, substrate engineering

## Abstract

Single-walled carbon nanotube (SWCNT) *p*–*n* junction thermoelectric generators develop an in-plane temperature difference under uniform heating. They convert it into a voltage with no cold source. This suits maintenance-free Internet-of-Things sensors. Two routes form such a junction and differ in how each region is processed, so which junction property governs the output has remained open. Here we compare a co-filtered route, which couples the *p*- and *n*-type regions in a single wet process, with carbon-paste bonding of two separately filtered films. A hyperbolic-tangent fit resolves each Seebeck profile into a transition width and a total Seebeck coefficient Δ*S*. Bonding preserves the intrinsic *p*-type coefficient. It raises Δ*S* from 76 to 98 µV/K and narrows the transition from 7.7 to below 3.5 mm. The internal resistance rises by 36–42%. On infrared-transparent cycloolefin polymer, the effective in-plane temperature difference differed by only 9% between the routes. The 29% gain in Δ*S* transferred directly to the output, 0.42 against 0.30 mV. The maximum power rose from 0.47 to 0.65 nW. The total Seebeck coefficient therefore sets the output. Bonding offers higher output and printable scalability; the co-filtered route offers a jointless film, higher electrical conductivity, mechanical robustness, and simpler processing.

## 1. Introduction

Internet of Things (IoT) sensor networks are expanding rapidly across infrastructure, healthcare, and logistics [1,2]. The need to replace batteries and to install wiring constrains their deployment [3,4]. Thermoelectric generators (TEGs) convert thermal energy directly into electricity through the Seebeck effect. They therefore supply power continuously from environmental heat [5,6,7,8,9]. They are promising candidates for autonomous micropower sources [10,11,12]. Recent work extends the concept to flexible and wearable platforms [13,14]. Carbon nanotubes [15], and single-walled carbon nanotubes (SWCNTs) in particular [16,17], suit such applications. SWCNTs are lightweight and flexible [18,19]. They also exhibit a high Seebeck coefficient for a carbon material near room temperature [20,21,22,23,24].

Two obstacles have historically limited SWCNT TEGs. The first is to prepare air-stable *n*-type material. As-grown SWCNTs behave as *p*-type semiconductors because adsorbed oxygen molecules withdraw electrons [25,26]. *n*-type doping by surface charge transfer is also prone to degradation in air, which has motivated the design of air-stable dopants [27,28,29,30]. Our previous studies addressed this obstacle. *n*-type SWCNT films were doped with dimethyldioctadecylammonium chloride (DODMAC), a cationic surfactant. These films retained their *n*-type Seebeck coefficient in air for more than two years [31]. We subsequently optimized the dopant concentration for thermal durability and low thermal conductivity [32]. The second obstacle is more fundamental. A conventional TEG requires both a heat source and a cold source. Maintaining the cold source imposes cost and volume penalties that are prohibitive for compact, maintenance-free IoT nodes.

A generator can circumvent this constraint. It must develop a temperature difference within the element itself under uniform heating. SWCNT films absorb strongly over a broad wavelength range that includes the infrared. Vertically aligned SWCNT forests approach blackbody behavior [33]. SWCNT assemblies convert light into heat efficiently [34,35,36,37]. The *p*- and *n*-type regions carry Seebeck coefficients of opposite sign. The voltages on either side of the junction therefore add along the film. The film thereby converts any in-plane temperature gradient into a net voltage without an external cold source. Under uniform heating, the film loses heat preferentially at its ends. The central region that contains the junction therefore remains the warmest part of the film. Our group has demonstrated devices that operate on this principle under uniform sunlight [38] and in *p*–*i*–*n* configurations under uniform heating [39]. A related device floats on water and generates the gradient in *p*-type films by evaporative cooling of selected areas [40]. The voltage that such a device delivers depends on two spatial profiles at once: the Seebeck coefficient along the film, and the temperature that the same film sustains. The output follows from the overlap between them, which Section 3.5 formulates quantitatively. Most previous work has addressed the first of these two profiles. It steepened d*S*/d*x* through the deposition interval during vacuum filtration [41], or patterned *p*–*n* regions in planar carbon nanotube devices [42]. The supporting layer rather than the film governs the second profile. Fewer studies have examined it, even though materials selection governs thermal management in electronics [43].

The substrate defines the thermal environment for such a comparison. Our group recently showed that the infrared absorptance and the thermal inertia of the substrate control the transient polarity of these devices [44]. The substrate also governs the steady-state temperature field through its thermal coupling to the heat source. Polyimide (PI) and cycloolefin polymer (COP) bracket the optical range that governs this coupling. Molecular absorption reduces the transmittance of PI across the whole fingerprint region, from 5 to 20 µm [45]. That region contains the 8.97 µm emission peak of a 323 K blackbody. COP is a non-polar cyclic olefin, and its strong absorptions lie near 3.4 µm. COP therefore transmits the band that PI absorbs. The blackbody-weighted transmittance at 323 K is 9% for PI and 61% for COP (Section 3.3). Their areal thermal resistances differ only by a factor of two. We therefore build every device on both substrates. The junction comparison then holds in an absorbing and in a transparent thermal environment.

In this work, we compare the two routes that form the SWCNT *p*–*n* junction. We identify which device property each route governs. The first route forms a co-filtered junction. We deposit the *p*- and *n*-type dispersions sequentially onto a single membrane filter [38,41]. One wet process and one reducing heat treatment therefore couple the two regions. The second route is modular. We filter the *p*- and *n*-type films separately and subsequently bond them with a conductive carbon paste [46,47]. This route exploits the modularity of freestanding SWCNT films [48]. We fit a hyperbolic tangent to each Seebeck coefficient profile. The fit characterizes the junction by a transition width *w* and a total Seebeck coefficient Δ*S*. The conventional slope metric d*S*/d*x* conflates these two quantities. We fabricate generators that carry the two junction types on PI and COP substrates. We heat them uniformly from beneath the substrate, without a cold source. We then compare the maximum power delivered to a matched load and the effective in-plane temperature difference Δ*T*_eff_ = *V*/Δ*S*. The comparison shows that the total Seebeck coefficient of the junction sets the output of a cold-source-free device. The choice between the two routes then reduces to a trade-off. Output and printable scalability favor bonding. Mechanical robustness and process simplicity favor the co-filtered route.

Two contributions distinguish this work from previous studies of *p*–*n* patterned nanotube films. The first is analytical. The hyperbolic-tangent fit resolves a junction into a transition width *w* and a total Seebeck coefficient Δ*S*, which the conventional slope metric d*S*/d*x* combines. The second is a design rule. Under uniform heating, Δ*S* rather than d*S*/d*x* sets the output. Section 3.6 places the resulting device metrics beside those reported for printed and patterned nanotube generators, and states the limitations of the present approach.

## 2. Materials and Methods

**Figure 1:**
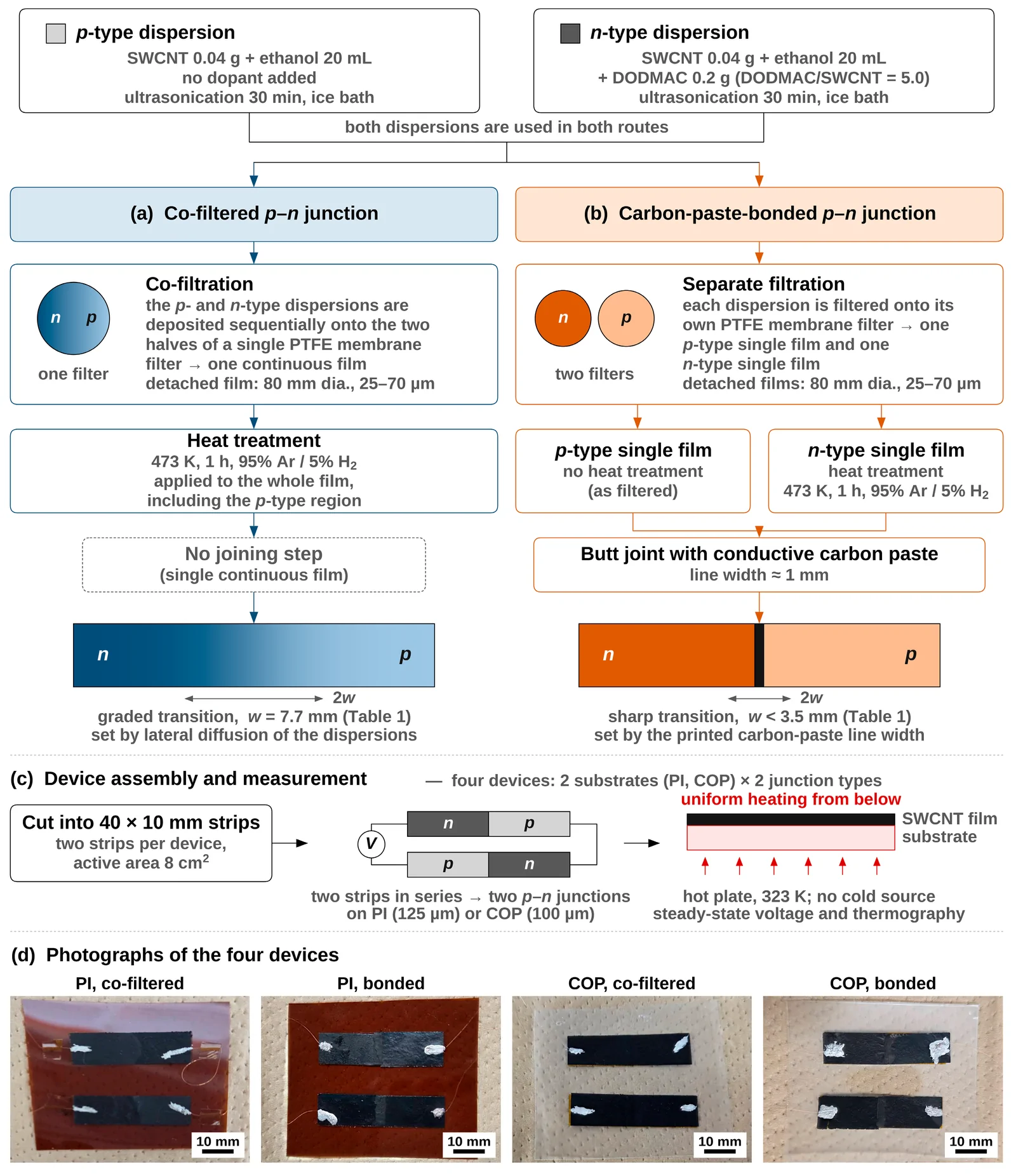
Fabrication routes for the two junction types and the device architecture. The *p*-type and *n*-type dispersions are prepared from the same SWCNT/ethanol base, and both dispersions are used in both routes. (**a**) Co-filtered *p*–*n* junction: the two dispersions are deposited sequentially onto the two halves of a single membrane filter, which gives one continuous film, and the reducing heat treatment (473 K, 1 h, 95% Ar and 5% H_2_) is applied to the whole film, including the *p*-type region. Lateral diffusion of the dispersions sets the graded transition (*w* = 7.7 mm, Table 1). (**b**) Carbon-paste-bonded *p*–*n* junction: each dispersion is filtered onto its own membrane; only the *n*-type single film receives the heat treatment, and the two films are then butt-joined along a conductive carbon paste line approximately 1 mm wide (*w* < 3.5 mm, limited by the measurement resolution). (**c**) Device assembly and measurement: each junction film is cut into strips of 40 mm × 10 mm, and two strips whose *p*- and *n*-type regions lie in opposite senses are connected in series on a PI (125 µm) or COP (100 µm) substrate, so that each device contains two junctions and an active area of 8 cm^2^. The four devices comprise two substrates × two junction types. The devices are heated uniformly from below at 323 K with no cold source. (**d**) Photographs of the four devices: from left to right, the co-filtered and carbon-paste-bonded devices on PI, followed by those on COP. Each panel carries a 10 mm scale bar. Blue and orange identify the co-filtered and the carbon-paste-bonded route, respectively, throughout this paper; within each route, the darker and the lighter tone denote the *n*-type and the *p*-type region.

Figure 1 summarizes the fabrication routes for the two junction types and the device architecture. The same pair of dispersions serves both routes. SWCNT powders (ZEONANO SG101, Zeon Corporation, Tokyo, Japan), synthesized by the super-growth method, were used as received. For the *p*-type dispersion, 0.04 g of SWCNTs was mixed with 20 mL of ethanol (FUJIFILM Wako Pure Chemical Corporation, Osaka, Japan). No dopant was added, since SWCNTs behave as *p*-type in air. For the *n*-type dispersion, 0.2 g of DODMAC (FUJIFILM Wako Pure Chemical Corporation, Osaka, Japan) was added to the same base composition. This corresponds to a DODMAC-to-SWCNT mass ratio of 5.0, within the range examined in [32]. An ultrasonic homogenizer (Sonifier 250, Branson Ultrasonics, Brookfield, CT, USA) dispersed each mixture at an output of 60% (nominal power 200 W) for 30 min in an ice bath. Vacuum filtration onto a polytetrafluoroethylene membrane filter (90 mm diameter, Advantec Toyo Kaisha, Tokyo, Japan) then formed freestanding films. The films were dried and detached. They measured 80 mm in diameter and 25–70 µm in thickness. The dispersed SWCNT mass set the thickness.

Two junction types were prepared from these dispersions. For the co-filtered *p*–*n* junction, the *p*-type and *n*-type dispersions were deposited onto the two halves of a single membrane filter (Figure 1a). The two regions merged into one continuous film. The dispersions diffuse laterally during filtration and thereby form a graded transition zone at the boundary [41]. For the carbon-paste-bonded junction (Figure 1b), a *p*-type single film and an *n*-type single film were filtered on separate membranes. The co-filtered *p*–*n* junction films and the *n*-type single films were then heat-treated at 473 K for 1 h in a reducing atmosphere (95% Ar and 5% H_2_). This treatment activates and stabilizes the *n*-type doping [31]. It reaches the whole co-filtered film, including its *p*-type region, whereas the *p*-type single films used for the bonded junctions were not heat-treated. Only after this treatment did a conductive carbon paste (REXALPHA RA FS 090 S, Toyochem, Tokyo, Japan) butt-join the *p*-type and *n*-type single films along a line approximately 1 mm wide.

Each junction film was cut into strips of 40 mm × 10 mm (Figure 1c). Throughout this paper, film denotes the freestanding membrane obtained by filtration and, in general statements, the SWCNT material itself. Strip denotes the 40 mm × 10 mm piece cut from a junction film. We use strip wherever the device geometry is at issue. Every device carries two such strips connected in series. The *p*- and *n*-type regions lie in opposite senses on the two strips, so that the two junction voltages add. Each device therefore has a total active area of 8 cm^2^ and two *p*–*n* junctions. This is the same series configuration as in our previous substrate study [44]. We report voltages as measured across the device. Where the analysis requires it, we divide them by two to give per-junction values. The strips were attached to one of two substrates: polyimide (PI; Kapton, DuPont, Wilmington, DE, USA; 125 µm thick, 60 × 50 mm) and cycloolefin polymer (COP; ZEONOR ZF16-100, Zeon Corporation, Tokyo, Japan; 100 µm thick, 60 × 50 mm). The two substrates differ in thickness and in material. Section 3.3 therefore compares them by areal thermal resistance rather than by thermal conductivity alone. This procedure yielded four devices (two substrates × two junction types). Figure 1d shows photographs of the four devices. The carbon-paste line appears as a band near the center of each bonded strip. One device of each combination was fabricated and measured. Section 3.4 describes the internal-consistency checks that support this single-device comparison. The two devices of a given junction type carry strips cut from the same junction film. The film morphology is therefore common to the PI and the COP devices. The substrate changes the thermal and the optical environment alone.

**Table 1:** Junction parameters obtained from the hyperbolic-tangent fits of the Seebeck coefficient profiles.

Junction Type	*S*_n_ (µV/K)	*S*_p_ (µV/K)	Δ*S*_total_ (µV/K)	*x*_0_ (mm)	*w* (mm)	*R* ^2^
Co-filtered	−49.7 ± 2.7	+26.6 ± 2.8	76 ± 4	0.2 ± 0.7	7.7 ± 1.3	0.992
Carbon-paste-bonded	−47.0 ± 1.4	+51.5 ± 1.6	98 ± 2	1.6 ± 0.3	<3.5	0.998

Field-emission scanning electron microscopy (FE-SEM; S-4800, Hitachi, Tokyo, Japan) resolved the surface morphologies of the films and of the carbon-paste joint. Tensile tests (MX-1000N-FA, Imada, Toyohashi, Japan) measured the mechanical properties. The specimens were scaled to one-tenth of the No. 13B geometry of JIS Z 2241, as in our previous work on SWCNT films [49]. This standard is the Japanese equivalent of ISO 6892-1. The parallel portion measured 1.25 mm in width and 5.0 mm in gauge length. The carbon-paste-bonded film could not be cut to this geometry without damaging the joint, so it was not tensile-tested. Two K-type thermocouples measured the in-plane Seebeck coefficient. They were pressed onto the film at a 5-mm separation. The measurement covered nine positions at 5-mm intervals, that is, a 40-mm span along the longitudinal axis of each junction film. The junction lay at the origin, and the localized temperature difference was 1–4 K. The four-point probe method (RT-70V, Napson, Tokyo, Japan) [50] measured the in-plane electrical conductivity at the corresponding positions. A micrometer (DM025, AS ONE, Osaka, Japan) measured the film thicknesses. The uncertainty of both transport quantities follows from nine *p*-type films prepared under identical conditions. Their Seebeck coefficient averages 53.5 µV/K with a standard deviation of 1.9 µV/K, that is, 3.5% of the mean. Their electrical conductivity averages 28.9 S/cm with a standard deviation of 3.3 S/cm, that is, 11.4% of the mean. The vertical error bars of the Seebeck coefficient and electrical conductivity profiles in Section 3.2 denote ±1.9 µV/K and ±11.4% of the local conductivity, respectively. They therefore combine the reproducibility of the preparation with that of the measurement. The conductivity scatters more widely because *σ* = 1/(*R*_s_*t*) contains the film thickness, which differs between films. The Seebeck coefficient is independent of the film thickness. The horizontal error bars of the Seebeck coefficient profiles denote the 5-mm separation of the two thermocouples. Fourier-transform infrared spectroscopy (IRSpirit-T, Shimadzu, Kyoto, Japan) recorded the infrared transmittance *T* of the SWCNT films and the two substrates over 400–4000 cm^−1^ (2.5–25 µm). This range contains the 8.97 µm emission peak of a 323 K blackbody source. We report the result below as the absorbance *A* = −log_10_*T*.

The devices were operated under uniform heating with no cold source. Each device rested on a hot-plate stirrer (iSTIR HP320, Neuation Technologies, Gandhinagar, India) set to a controller temperature of 323 K. The stirrer heated the device from beneath the substrate. A data logger (LR8432, Hioki E.E., Ueda, Japan) recorded the output voltage and the device temperature. A thermographic camera (Xi 400, OPTXI40LTF20CFKT090, Optris, Berlin, Germany) imaged the surface temperature distribution of the device. It sampled three zones along each strip: the two ends and the central region that contains the junction. The device temperature rise quoted below refers to the central zone, referenced to its own pre-heating value. The voltage was recorded from 5 min before the heater was switched on until 10 min afterward. Throughout this paper, we analyze only the steady-state output, defined as the output voltage averaged over the last minute of the heating period. The transient response immediately after the heater is switched on includes an initial negative voltage excursion. The infrared absorptance and the thermal inertia of the substrate govern that response. We have reported and modeled it separately [44]. A digital multimeter (R6561, Advantest, Tokyo, Japan) measured the internal resistance *R*_int_ of each device at steady state under the same heating conditions. The measurement used the two-terminal method across the SWCNT films. We calculated the maximum power as *P*_max_ = *V*^2^/(4*R*_int_), the power delivered to a matched load. Here, *V* is the steady-state output voltage. Both quantities refer to the device as a whole, which contains two series-connected junctions.

## 3. Results and Discussion

### 3.1. Morphology and Mechanical Properties of the Junction Films

**Figure 2:**
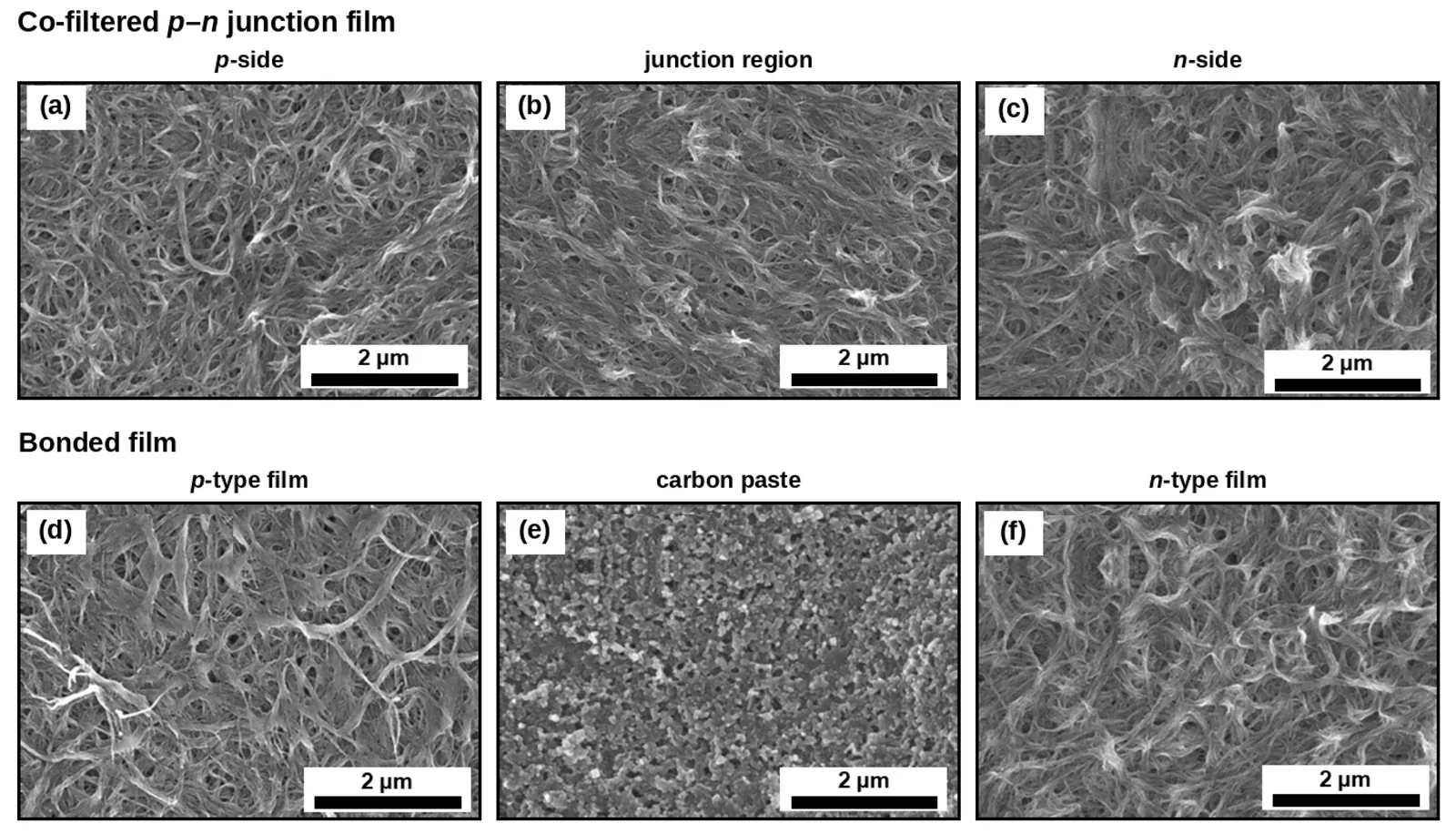
FE-SEM images of the junction films: (**a**) *p*-side, (**b**) junction region, and (**c**) *n*-side of the co-filtered *p*–*n* junction film; (**d**) *p*-type film, (**e**) carbon paste, and (**f**) *n*-type film of the carbon-paste-bonded junction. Each panel carries a 2 µm scale bar.

Figure 2 shows the FE-SEM images of the two junction types. The co-filtered film (Figure 2a–c) shows no discrete boundary, and the *p*- and *n*-type regions merge continuously. This is consistent with the graded transition zone that forms as the dispersions diffuse laterally during sequential filtration [41]. In the bonded film (Figure 2d–f), the carbon particles of the paste appear at the joint. They differ morphologically from the nanotube network on either side. In both films, the bundles in the *n*-type region appear thicker than those in the *p*-type region. We attribute this to DODMAC molecules that encapsulate the SWCNT bundles [31]. Our previous work characterized the coarsened *n*-type network and its effect on transport quantitatively [44].

Figure S1 (Supplementary Materials) summarizes the tensile properties of the *p*-type single, *n*-type single, and co-filtered *p*–*n* junction films. The three film types differed in strength and ductility (mean ± one standard deviation, *n* = 5). The *p*-type single film was the strongest and the most ductile (8.96 ± 2.20 MPa, 2.83 ± 1.03%). The *n*-type single film and the co-filtered film were both weaker and less ductile (6.73 ± 2.34 and 5.38 ± 2.10 MPa; 1.25 ± 0.47 and 1.10 ± 0.52%). The Young’s modulus moved in the opposite direction. It rose from 0.39 ± 0.08 GPa for the *p*-type film to 0.61 ± 0.14 and 0.60 ± 0.13 GPa for the *n*-type and co-filtered films (Figure S1d). Both trends follow from the structure that the surfactant imposes. DODMAC coarsens the bundle network and alters how load transfers between bundles. The carbon-paste joint could not be tested quantitatively because it failed while the tensile specimen was being cut. The joint is therefore mechanically weaker than the surrounding film.

### 3.2. Seebeck Coefficient Profile: Separating the Transition Width from the Total Seebeck Coefficient

**Figure 3:**
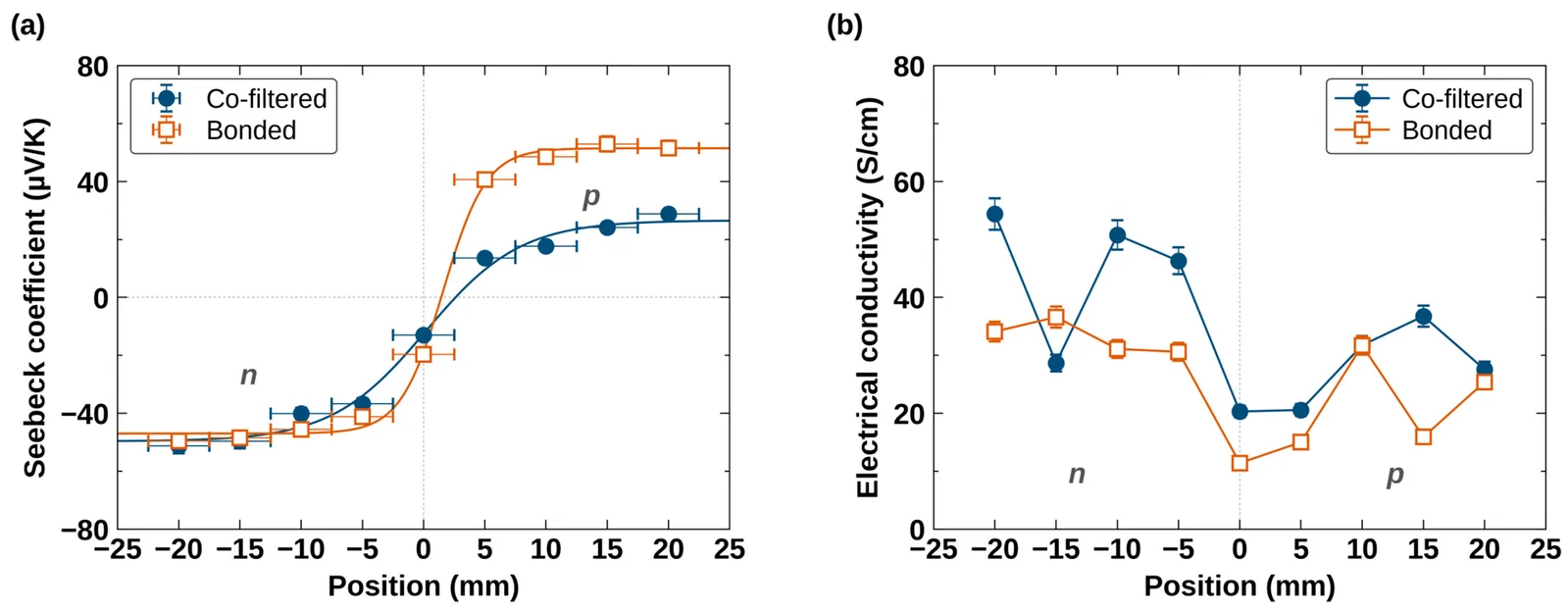
Spatial profiles of (**a**) the Seebeck coefficient with hyperbolic-tangent fits (solid lines) and (**b**) the electrical conductivity of the co-filtered and carbon-paste-bonded junction films, measured at 5-mm intervals with the junction at the origin. In (**a**), the vertical error bars denote ±1.9 µV/K, and in (**b**) they denote ±11.4% of the local conductivity. Both follow from nine *p*-type films prepared under identical conditions (Section 2). In (**a**), the horizontal error bars denote the 5-mm separation of the two thermocouples. The internal resistances quoted in the text follow from integrating the reciprocal of the conductivity profile in (**b**) over the strip geometry, and therefore refer to the SWCNT material alone.

Both routes form a continuous *p*–*n* junction. Figure 3a shows the spatial profiles of the Seebeck coefficient across the two junction films, measured at 5-mm intervals with the junction at the origin. Both profiles vary monotonically from negative (*n*-type) to positive (*p*-type) values. We fitted a hyperbolic-tangent function to each profile:

(1) *S*(*x*) = (*S*_p_ + *S*_n_)/2 + [(*S*_p_ − *S*_n_)/2]tanh[(*x* − *x*_0_)/*w*].

The fit defines the plateau values *S*_p_ and *S*_n_, the junction position *x*_0_, and the transition width *w*. Here *w* is the characteristic length of the hyperbolic tangent, so the 10–90% extent of the transition spans approximately 2.2*w*. The fits describe both data sets with *R*^2^ > 0.99, and Table 1 lists the extracted parameters. This function separates two quantities that the conventional maximum-slope metric d*S*/d*x* = Δ*S*/2*w* combines. A film shows a large slope for either of two reasons: a spatially sharp junction (small *w*), or a large total Seebeck coefficient Δ*S*_total_ = *S*_p_ − *S*_n_. We write Δ*S* for this quantity below. The two quantities arise from different mechanisms, so we report them separately. Figure S2 (Supplementary Materials) plots the residuals of the two fits against position. Applying the parameters of Table 1 to the measured values of Figure 3a gives a root-mean-square residual of 2.8 µV/K for the co-filtered profile and 1.9 µV/K for the bonded profile, that is, 3.7% and 2.0% of the corresponding Δ*S*.

Figure 3b shows the corresponding electrical conductivity profiles. In both films, the *n*-type region conducts better than the *p*-type region, consistent with electron donation by the dopant [27]. A local minimum appears at the joint of the bonded film. The conductivity there falls to 11 S/cm, versus 15–37 S/cm at the eight other positions along the film. In the co-filtered film, the dip near the junction does not exceed the scatter along the film. The Seebeck coefficient at the joint of the bonded film is negative (−20 µV/K), so the carbon paste itself conducts as an *n*-type material. The joint is therefore a short *n*-type segment inserted between the two films, not a simple *p*–*n* interface. This segment is only about 1 mm wide. Away from the joint, the co-filtered film conducts better. Integrating the reciprocal of the conductivity profile over the strip geometry gives the resistance of the SWCNT material alone. That resistance is 44% higher for the bonded junction. The two bonded devices measured 68 Ω and the two co-filtered devices 48–50 Ω. That is 36% to 42% higher (Section 3.4). The same films scatter by 11.4% in the electrical conductivity (Section 2). The resistance difference therefore also exceeds the film-to-film variation. The bonded route therefore raises the total Seebeck coefficient and the internal resistance together. Section 3.4 weighs the two against each other in terms of the delivered power.

The uncertainty in Δ*S*_total_ is propagated from the plateau uncertainties. The transition width of the bonded junction is an upper bound. Each Seebeck value averages the profile over the 5-mm separation of the two thermocouples, which broadens the apparent transition. The fitted value of 3.5 ± 0.4 mm therefore bounds the true width from above. The root-mean-square residuals of the two fits, obtained by applying these parameters to the measured values of Figure 3a, are 2.8 and 1.9 µV/K for the co-filtered and the bonded junction. Figure S2 plots them against position.

**Figure 4:**
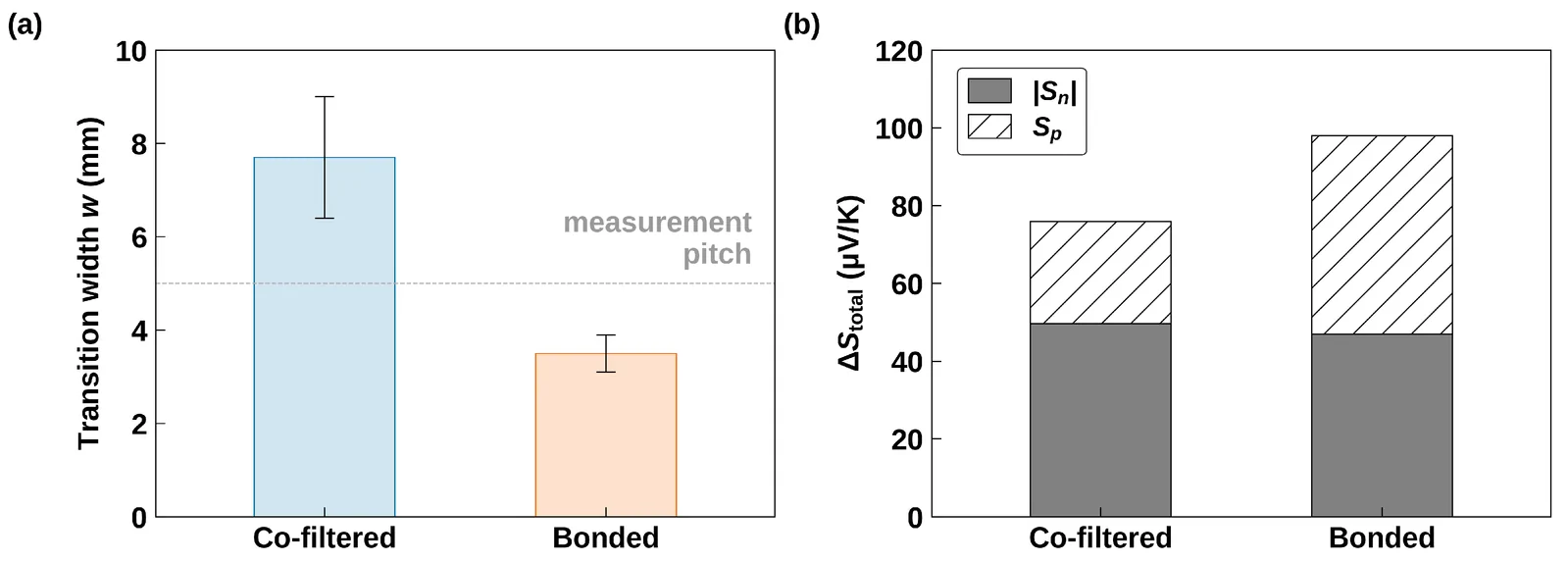
Junction parameters extracted from the fits in Figure 3a: (**a**) transition width *w*, and (**b**) total Seebeck coefficient Δ*S*_total_ decomposed into the |*S*_n_| and *S*_p_ plateau contributions. In (**a**), the dashed line marks the 5-mm thermocouple separation. The bonded bar shows the fitted value, which bounds the true width from above (Section 3.2).

The co-filtered junction has a transition width *w* = 7.7 ± 1.3 mm (Figure 4a). This value agrees in order of magnitude with the lateral diffusion distance of the dispersions in the wet film during sequential filtration. The masking geometry and the wet process reproduce this graded interface [41]. The fit to the carbon-paste-bonded junction instead gives *w* = 3.5 ± 0.4 mm. This fitted value constitutes an upper bound on the true width. The reason is instrumental: each Seebeck value averages the profile over the 5-mm separation of the two thermocouples. This averaging window broadens the apparent transition. We therefore quote *w* < 3.5 mm. The same window affects the co-filtered profile only weakly, because its width exceeds the window. The 5-mm separation of the two thermocouples sets this resolution, and the 5-mm scanning step does not. A finer scan therefore leaves the averaging window unchanged. A potential-Seebeck microprobe resolves this length scale, since the temperature field of its heated tip extends over tens to a few hundred micrometers in thin films [51]. The upper bound on *w* leaves the conclusions unchanged, because Δ*S* sets the output of the device (Section 3.5). The true transition width of the bonded junction should approach the 1 mm paste line width. The two routes differ in what sets this width. In the co-filtered route, a material length scale fixes it: the lateral diffusion of the dispersion. This length does not scale down with the device. In the bonded route, a process parameter fixes it: the printed line width. Carbon-ink printing could narrow it further [46,47]. The fitted junction position of the bonded film is *x*_0_ = 1.6 ± 0.3 mm. The joint therefore sits slightly off the nominal center of the strip.

The two routes also differ in the total Seebeck coefficient. The *n*-side plateaus of the two films nearly coincide (−49.7 ± 2.7 versus −47.0 ± 1.4 µV/K). These two films passed through different routes, and their *n*-side plateaus agree to within 5.5%. The reproducibility of the *n*-type region is therefore comparable to the 3.5% scatter reported in Section 2. The *p*-side plateaus differ: +26.6 ± 2.8 µV/K for the co-filtered film compared with +51.5 ± 1.6 µV/K for the bonded film (Figure 4b). By construction, the bonded *p*-side plateau is that of an undoped film that bypassed the heat treatment. It represents the intrinsic *p*-type coefficient directly. The co-filtered value is approximately half of it. We attribute this difference to two features of the co-filtered route. DODMAC migrates laterally into the *p*-type region during filtration. The reducing heat treatment at 473 K exposes the entire co-filtered film, including the *p*-type region. This treatment activates the *n*-type doping [31,32]. In the bonded route, each film passes through its own optimal conditions before joining, so the *p*-type region retains its intrinsic properties. The two halves of a co-filtered film cannot pass through different treatments. Bonding instead joins the films only after each has completed its own process. Δ*S* consequently increases from 76 ± 4 to 98 ± 2 µV/K. Nine nominally identical films scatter by 3.5% in the Seebeck coefficient (Section 2). This 29% increase therefore does not arise from film-to-film variation. The two routes therefore complement each other. Carbon-paste bonding raises the total Seebeck coefficient and sharpens the transition, whereas the co-filtered route retains the higher electrical conductivity.

### 3.3. Thermal and Optical Properties of the Substrates

Figure S3 (Supplementary Materials) summarizes the thermal conductivities and the sheet thermal conductances of the SWCNT films and the two substrates. Figure S4 shows their infrared absorbance spectra. Table 2 lists the values that enter the device analysis. All thermal conductivities are reference values rather than measurements of the present specimens. Our previous substrate study measured the SWCNT values for films prepared by the same route [44]: 2.9 W/(m·K) in-plane for the *p*-type film and 3.9 W/(m·K) for the *n*-type film. Both values lie in the range reported for randomly oriented SWCNT networks [52,53]. In such networks, the inter-tube junctions limit heat transport rather than the intrinsic tube conductivity. The higher *n*-type value parallels the coarser, more strongly aggregated bundle network that DODMAC encapsulation produces (Figure 2). The substrate values, 0.12 W/(m·K) for PI and 0.20 W/(m·K) for COP, are nominal figures for the commercial films used here. The substrates are unfilled polymer films, so we treat them as thermally isotropic.

Two conclusions from these numbers constrain the device analysis. First, the SWCNT films have a sheet thermal conductance of *κt* ≈ 0.7–2.7 × 10^−4^ W/K at the thicknesses used here. This value exceeds that of either substrate by a factor of 4 to 18: 1.5 × 10^−5^ W/K for PI and 2.0 × 10^−5^ W/K for COP (Figure S3b). The SWCNT film therefore spreads heat laterally within the device, and the substrate does not. The substrate instead couples the device vertically to the heat source through its areal thermal resistance *t*/*κ*. This resistance amounts to 1.0 × 10^−3^ m^2^·K/W for PI and 5.0 × 10^−4^ m^2^·K/W for COP. The PI devices present twice the areal resistance. Vertical conduction alone would therefore let them sustain the larger in-plane temperature difference. The areal thermal resistance, however, describes only how heat reaches the device, and not where the incident radiation is absorbed. A second parameter is therefore required. Second, the infrared response supplies that parameter and separates the two substrates cleanly (Figure S4). The measured range of 2.5–25 µm contains the 8.97 µm emission peak of the 323 K source. We report the spectra as absorbance *A*, from which the transmittance follows as *T* = 10^−^*^A^*. The SWCNT films are opaque throughout the range (*A* ≥ 2.0, transmittance of about 1% or less), so they absorb the radiation that reaches them. PI absorbs at the emission peak: *A* = 2.4–3.6 over 8.5–9.5 µm. COP instead shows *A* = 0.25 there and remains below about 0.3 over most of the range, interrupted only by narrow absorption bands. Weighting the transmittance by the 323 K blackbody spectrum gives effective transmittances of 9% for PI and 61% for COP. Table 2 carries these two numbers into the device analysis. PI therefore absorbs the incident radiation largely within the substrate. COP passes the larger part of it to the SWCNT film. The ordering agrees with the single-wavelength transmittances at 8.8 µm of our previous report (COP 45.8%, PI 0.15%) [44], although the broadband weighting shifts the absolute values.

### 3.4. Steady-State Device Performance Under Uniform Heating

**Figure 5:**
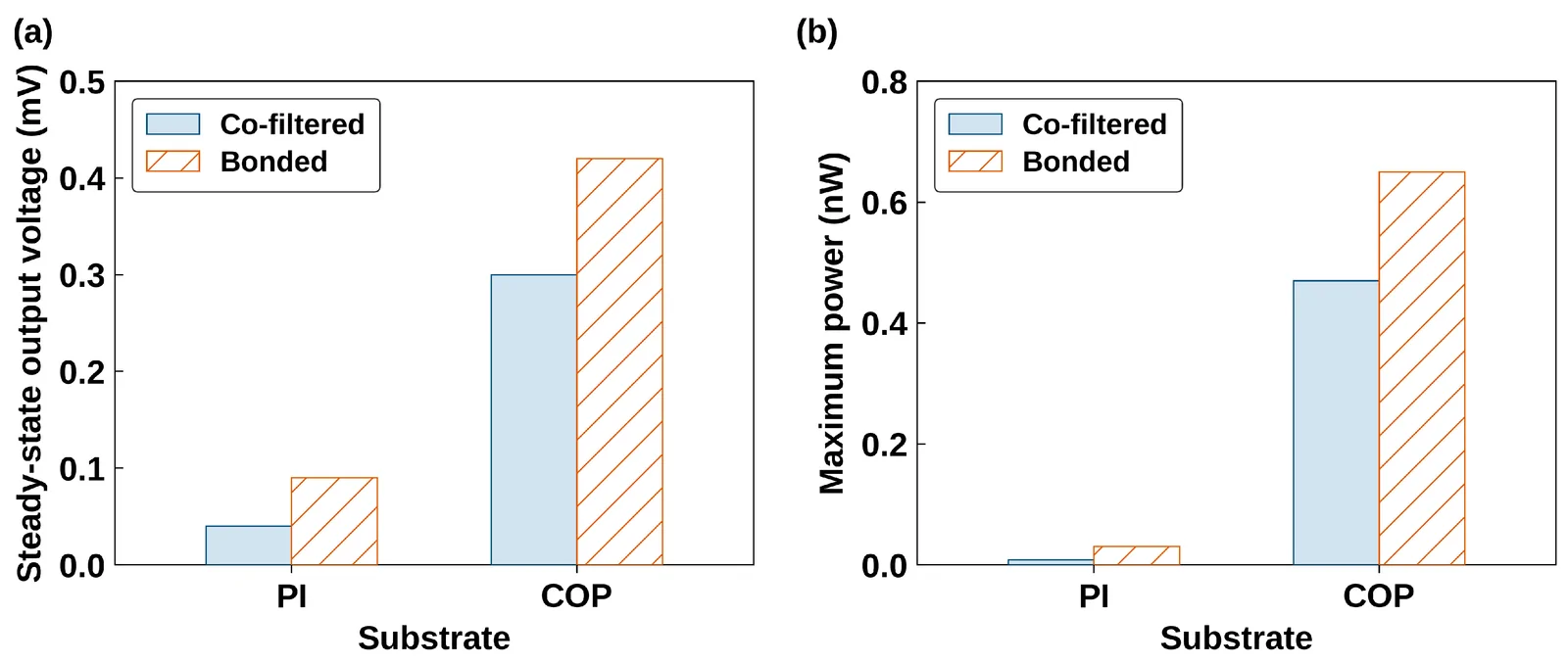
(**a**) Steady-state output voltages and (**b**) maximum powers of the four devices (two substrates × two junction types) under uniform heating from below. Both quantities refer to the device, which contains two series-connected junctions. Maximum power is the power delivered to a matched load: *P*_max_ = *V*^2^/(4*R*_int_).

All four devices generated a positive steady-state voltage with no cold source. This confirms that the *p*–*n* junction architecture sustains a self-generated in-plane temperature difference under uniform heating [38,39]. Figure 5a shows the steady-state output voltages of the four devices, and Figure 5b shows the corresponding maximum powers. Table 2 summarizes the values. Figure S5 (Supplementary Materials) gives the complete voltage transients from which we extracted them. One device of each combination was measured. Two independent consistency checks support the comparison. The measured device resistances agree with those predicted by integrating each film’s conductivity profile (Section 3.2). Scaling the co-filtered output by the ratio of the fitted total Seebeck coefficients reproduces the bonded output on COP to within 8% (Section 3.5). Changing the substrate from PI to COP raised the output from 0.04 to 0.30 mV for the co-filtered junction and from 0.09 to 0.42 mV for the bonded junction. Changing the junction route on a fixed substrate raised it from 0.30 to 0.42 mV on COP and from 0.04 to 0.09 mV on PI. The maximum powers follow the same ordering. On COP, the bonded device delivered 0.65 nW versus 0.47 nW for the co-filtered device, 38% more. On PI, it delivered 0.030 nW compared with 0.008 nW. The substrate therefore dominates. It changes the output voltage by a factor of five to eight and the delivered power by a factor of twenty to sixty. The junction route changes the voltage by 40% and the power by 38% on COP. On PI, it changes the voltage by about a factor of two and the power by about a factor of four, and both outputs there are small.

The two junction routes also differ in internal resistance, and this difference acts on the delivered power alone. The bonded devices measured *R*_int_ = 68 Ω and the co-filtered devices 48–50 Ω. The bonded route is therefore 36% to 42% higher, depending on the substrate. Integrating the conductivity profile gives 44% (Section 3.2). The junction route sets the internal resistance, and the substrate does not. The co-filtered devices measured 48 Ω on COP and 50 Ω on PI, and both bonded devices measured 68 Ω (Table 2). The higher output of the COP devices therefore follows from their larger in-plane temperature difference, and not from a change in resistance. These values refer to the SWCNT material. An external load additionally sees the contact and interconnect resistances, which limit the output of printed thermoelectric devices generally [54]. The Seebeck coefficient and the temperature field set the output voltage. The internal resistance does not affect it. The internal resistance enters only through the power delivered to a matched load, *P*_max_ = *V*^2^/(4*R*_int_). That power scales as *V*^2^/*R*_int_. The bonded device on COP has a 40% higher voltage and a 42% higher internal resistance (68 versus 48 Ω). These values predict a power ratio of 1.40^2^/1.42 = 1.38. We observed a gain of 38%. The resistance penalty of the bonded route therefore does not offset its gain in the total Seebeck coefficient. The maximum power is computed from the measured steady-state voltage and the measured internal resistance. Figure 5b therefore combines the two measurements of Figure 5a and Table 2. It does not report a separate measurement of the delivered power.

The higher electrical conductivity of the co-filtered route therefore does not increase the delivered power greatly. That route retains, in addition, a mechanical advantage. Its continuous film contains no discrete joint, survived cutting into tensile specimens (Section 3.1), and forms in a single wet process. The co-filtered route therefore offers mechanical robustness and process simplicity, which the bonded route does not provide.

### 3.5. Effective In-Plane Temperature Difference and the Role of the Substrate

**Figure 6:**
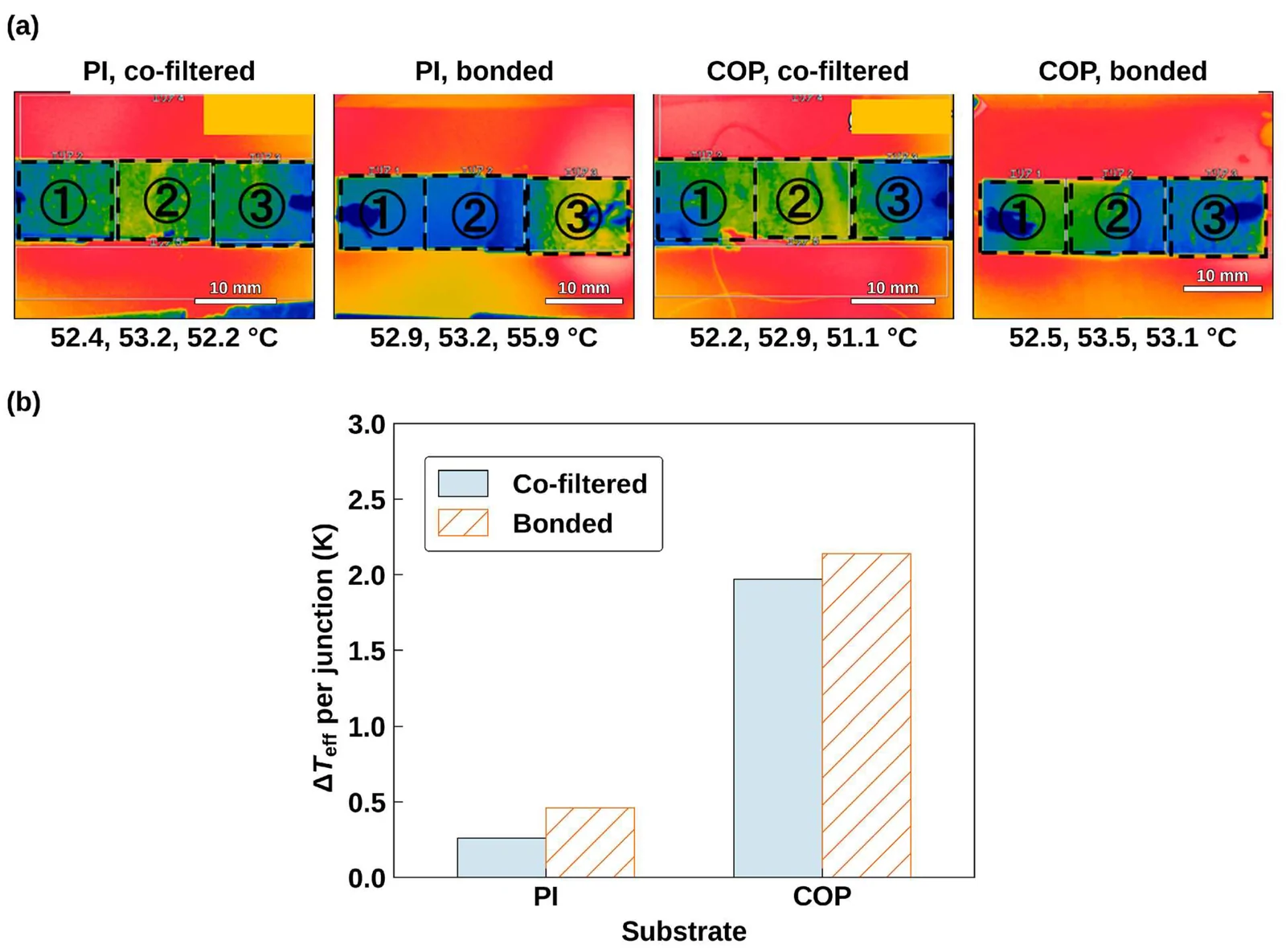
(**a**) Thermographic images of the strip surfaces at steady state for the four devices, with the three zones marked along each strip. Each panel carries a 10 mm scale bar. The values below each image are the absolute steady-state zone temperatures, in °C, for zones 1, 2, and 3. The center-minus-end differences compiled in Table 2 follow directly from them. The center-zone temperature rise in Table 2 is instead referenced to each device’s pre-heating temperature. These absolute values alone do not give it. Zone 2 contains the junction. The color scale is auto-ranged independently for each image. The panels should therefore be compared through the quoted zone temperatures, not through color. (**b**) Effective in-plane temperature difference per junction, Δ*T*_eff_ = *V*/Δ*S*, for the four devices, grouped by substrate. Solid and hatched bars denote the co-filtered and carbon-paste-bonded devices, respectively. On COP, the two routes give nearly equal values, whereas the two substrates differ by a factor of four to eight. Table 2 lists the values.

Figure 6a shows representative thermographic images of the strip surfaces at steady state, with the three zone temperatures quoted below each image. The center zone, zone 2, contains the junction. The three zone temperatures along the strip spread by 1.0–3.0 K in all four devices. In the co-filtered devices, the center zone exceeded the mean of the two end zones by 0.90 K on PI and by 1.25 K on COP. The bonded device on COP behaved in the same way, its center zone exceeding the mean of the ends by 0.70 K. The bonded device on PI forms the exception. Its center zone fell 1.20 K below the mean of the ends, and the device still produced a positive output. Its thermographic image was the most spatially heterogeneous of the four. The three-zone sampling resolves the magnitude and the ordering of the temperature field. It does not resolve the local profile near the junction, where the Seebeck coefficient varies most steeply. The integral formulation below weights the temperature field by d*S*/d*x*. For the bonded junction, half of that weight lies within 1.9 mm of the joint. Each thermographic zone averages over a much longer section of the strip. The local sign reversal on PI therefore does not contradict the positive output. Both PI outputs also lie within a few times the baseline fluctuation of the voltage record (Figure S5).

To place the output voltages on a common thermal footing, we calculated the effective in-plane temperature difference Δ*T*_eff_ = *V*/Δ*S* for each device. We used the per-junction voltage and the fitted total Seebeck coefficient of the corresponding junction film (Table 1). The open-circuit voltage follows from the overlap between the Seebeck coefficient profile *S*(*x*) and the temperature profile *T*(*x*):

(2) *V*_oc_ = −∫*S*(*x*)(d*T*/d*x*)d*x* = ∫(d*S*/d*x*)[*T*(*x*) − *T*_end_]d*x*.

The second form follows by integration by parts, provided that both ends of the strip lie at the same temperature *T*_end_. Substituting the fitted profile of Equation (1) gives d*S*/d*x* = (Δ*S*/2*w*)sech^2^[(*x* − *x*_0_)/*w*]. Equation (2) therefore reads *V*_oc_ = Δ*S*∫g(*x*)[*T*(*x*) − *T*_end_]d*x*, with the weight g(*x*) = (1/2*w*)sech^2^[(*x* − *x*_0_)/*w*]. This weight integrates to unity along the strip. Δ*T*_eff_ = *V*/Δ*S* is therefore the g-weighted mean of the temperature excess, and not an approximation to it. The weight is concentrated at the junction. Its cumulative fraction within |*x* − *x*_0_| ≤ *a* equals tanh(*a*/*w*). Half of the output therefore arises within 0.55*w* of the junction, and nine tenths within 1.47*w*. These half-widths are 4.2 and 11.3 mm for the co-filtered junction. They are at most 1.9 and 5.2 mm for the bonded junction, because *w* is an upper bound there. All of these lengths lie inside the 40-mm span over which the Seebeck profile was measured.

The zone temperatures of Figure 6a are consistent with this normalization in order of magnitude. The measured spread of 1.0–3.0 K along the strip is of the same order as the Δ*T*_eff_ values of 0.26–2.14 K. For the co-filtered devices, the center-minus-end excess is larger on COP than on PI (1.25 versus 0.90 K), and so is Δ*T*_eff_ (1.97 versus 0.26 K). Δ*T*_eff_ can nevertheless exceed the measured zone spread, as it does for the bonded device on COP (2.14 K versus a 1.0 K spread). The weighting accounts for this. Δ*T*_eff_ averages the temperature excess over a few millimeters about the junction. Each thermographic zone averages over a much longer section of the 40-mm strip. The two quantities therefore sample different lengths. The thermographic temperatures also carry the uncertainty of the assumed emissivity. We therefore treat the thermography as a check on the magnitude and the ordering of the temperature field, and Δ*T*_eff_ as the quantitative measure.

Figure 6b compares Δ*T*_eff_ across the four devices. The substrate divides them most. Δ*T*_eff_ reaches 1.97 and 2.14 K on COP, compared with 0.26 and 0.46 K on PI, for nominally identical active films. The areal thermal resistance alone predicts the opposite ordering (Section 3.3). COP transmits the radiation, so the SWCNT film absorbs it directly and sustains the in-plane gradient. PI instead absorbs the radiation within the substrate and clamps the film close to the uniform source temperature. Both junction types therefore reproduce the optical mechanism we identified previously [44]. That mechanism establishes the thermal environment in which the two routes are compared below.

The junction route divides the devices less. On COP, Δ*T*_eff_ differs between the two routes by only 9% (2.14 versus 1.97 K). Δ*T*_eff_ normalizes the per-junction output by Δ*S*. This near-equality therefore shows that the two junction types sustain nearly the same in-plane temperature difference on the same substrate. The two routes weight that field over different lengths. Half of the weight lies within 4.2 mm for the co-filtered junction, and within at most 1.9 mm for the bonded junction. Their agreement within 9% therefore places an upper bound on how steeply the temperature varies over this range. The 29% higher total Seebeck coefficient of the bonded device explains its 40% higher output (0.42 versus 0.30 mV). Scaling the co-filtered output by the Δ*S* ratio alone predicts 0.39 mV, within 8% of the measured 0.42 mV. The total Seebeck coefficient of the junction therefore sets the output, rather than any difference in the temperature field. On PI, the bonded device again leads (0.09 versus 0.04 mV). Both PI outputs are small, so we base the comparison of the two routes on the COP devices.

A comparison with our previous results places these outputs in context. The best device of the present study, the carbon-paste-bonded junction on COP, delivered 0.21 mV per junction. The COP-based device of our previous substrate study delivered 0.19 mV per junction, that is, 0.38 mV from two series-connected junctions [44]. The present co-filtered COP device delivered 0.15 mV per junction. The best device delivered 0.65 nW from an active area of 8 cm^2^, that is, about 0.08 nW/cm^2^, under a source at 323 K. Both the open-circuit voltage and the internal resistance scale linearly with the number of series-connected junctions, so the maximum power scales linearly as well. More than 10^3^ junctions are therefore required to reach the microwatt input level of commercial boost converters from a 323 K source. A correspondingly larger in-plane temperature difference per junction would serve the same end. A practical device must increase the number of junctions and Δ*T*_eff_ together, and the substrate governs the latter. The bonded route admits the first of these, because a printed line width rather than a diffusion length defines its junction. Carbon-ink printing [46,47] could therefore write junction arrays at a pitch that the co-filtered route cannot reach.

### 3.6. Comparison with Reported Carbon Nanotube Thermoelectric Generators

The present devices trade absolute power for the absence of a cold source, and Table 3 makes that trade explicit. Two groups of studies frame the comparison. The first group defines the *p*–*n* pattern within a single film, and a recent review collects these strategies [55]. Plasma treatment reaches a resolution of 1–2 µm [56], photobase generators pattern the film through a photomask [57], and aerosol doping reaches 1 µm [58]. The transition width of the present bonded junction, below 3.5 mm, is three orders of magnitude coarser. The second group applies an external temperature difference and reports device-level power. A fully printed SWCNT generator delivers 459 µW at Δ*T* = 300 K [59]. A screen-printed origami generator of 143 thermoelements delivers 25.7 µW at Δ*T* = 60 K [60]. An SWCNT/copolymer generator delivers 117 nW at Δ*T* = 30 K [61]. The present bonded device on COP delivers 0.65 nW from two junctions with no cold source. Its 323 K source develops 2.1 K per junction within the film.

Three factors account for this ordering, and each identifies where the present architecture applies. The devices of the second group receive Δ*T* from an external cold side. The present device instead develops about 2 K within itself under uniform heating. The output power scales as Δ*T*^2^, so the temperature difference alone accounts for a factor of 10^2^ to 10^4^. The number of series-connected *p*–*n* pairs accounts for a further factor of about 36, since the origami device carries 143 thermoelements, that is, about 72 pairs [60]. The transition width does not enter this comparison. In a cold-source-free device, the output follows from Δ*S* and the temperature profile through Equation (2). The element length is set instead by the thermal decay length of the film, which lies in the millimeter range. A micrometer-scale junction therefore raises the output only when the thermal design shortens that decay length. The design rule that follows from Table 3 is to raise Δ*S* and the number of junctions, and to leave *w* where the thermal length scale places it.

Normalization removes the two factors that dominate this ordering. For a generator of *N* series-connected junctions, *P*_max_ = *N*Δ*S*^2^Δ*T*^2^/(4*R*_1_), where *R*_1_ is the resistance of one junction. The ratio *P*_max_/(*N*Δ*T*^2^) = Δ*S*^2^/(4*R*_1_) is therefore independent of the applied temperature difference and of the junction count. It characterizes the junction alone. The bonded device of the present study gives 0.071 nW/K^2^, and the co-filtered device gives 0.060 nW/K^2^. The screen-printed origami generator gives 0.10 nW/K^2^ for its 72 *p*–*n* pairs [60]. These three values lie within a factor of two of one another. The printed generator of [59] joins 168 single-type legs with silver paste and therefore contains no *p*–*n* pairs. The four orders of magnitude that separate the origami generator from the present devices therefore follow from Δ*T* and *N*. Two devices built from other materials belong to the second group. A printed lateral *p*–*n* junction of Bi_0.5_Sb_1.5_Te_3_ and Bi_2_Te_2.7_Se_0.3_ delivers 5.3 µW/cm^2^ at Δ*T* = 25 K from a single thermocouple [54]. A Zn-doped Sb_2_Te_3_ film generator delivers 1100 nW at Δ*T* = 40 K [62]. Both require an external cold side.

The present study has five limitations. The output lies in the nanowatt range, four to six orders of magnitude below the printed generators of Table 3. One device was built for each of the four combinations. The comparison of the two routes therefore rests on the internal-consistency checks of Section 3.4 rather than on a device population. The 5-mm thermocouple separation bounds the transition width of the bonded junction from above rather than resolving it. The carbon-paste joint is the mechanically weakest part of the bonded device, and it failed during tensile specimen preparation. *R*_int_ refers to the SWCNT material and excludes the contact and interconnect resistances, so the quoted maximum powers are upper estimates.

## 4. Conclusions

The total Seebeck coefficient of the junction sets the output of a cold-source-free SWCNT *p*–*n* junction generator. We established this by comparing two routes for forming the junction: the co-filtered route and carbon-paste bonding of two separately filtered films. We built both junctions on two substrates that bracket the relevant infrared range. Carbon-paste bonding lets each film complete its own optimal process before joining. It thereby preserves the intrinsic *p*-type Seebeck coefficient. It raises the junction total from 76 to 98 µV/K and narrows the transition width from 7.7 mm to below 3.5 mm. The co-filtered junction instead conducts better and avoids a discrete joint. Its continuous film survived cutting into tensile specimens, whereas the carbon-paste joint failed during that cutting. Under uniform heating without a cold source, the bonded junction delivered the higher steady-state output on both substrates (0.42 versus 0.30 mV on COP and 0.09 versus 0.04 mV on PI). It also delivered the higher maximum power (0.65 versus 0.47 nW on COP and 0.030 versus 0.008 nW on PI). It carries a 36–42% higher internal resistance. The higher voltage outweighs this penalty, because the power scales as *V*^2^/*R*_int_. We evaluated the effective in-plane temperature difference Δ*T*_eff_ = *V*/Δ*S* per junction and checked it against thermography. This quantity is the d*S*/d*x*-weighted mean of the temperature excess along the strip. On COP, it differed between the two routes by only 9% (2.14 versus 1.97 K). The 29% gain in the total Seebeck coefficient therefore transferred directly to the output. The substrate, by contrast, set the magnitude of Δ*T*_eff_. The infrared-transparent COP sustained 1.97–2.14 K, compared with 0.26–0.46 K for the absorbing PI.

The choice between the routes depends on which requirement is decisive. Output and printable scalability favor bonding, whereas mechanical robustness and process simplicity favor the co-filtered route. Neither route is a finished process, and the present comparison indicates where each can improve. In the co-filtered route, two factors reduce the *p*-type Seebeck coefficient to approximately half of its intrinsic value: lateral migration of the dopant and exposure of the whole co-filtered film to the reducing heat treatment. A diffusion length sets the transition width. Masking the *p*-type region during filtration and restricting the heat treatment to the *n*-type half would address both limitations. In carbon-paste bonding, the joint forms the weakest point of the device. It is weak mechanically because it failed during specimen cutting. It is weak electrically, because the conductivity falls locally and the paste acts as a short *n*-type segment. Printing carbon inks of higher conductivity and stronger adhesion would address both weaknesses. Such printing would also narrow the transition toward the printed line width. The absolute power is about 0.08 nW/cm^2^ under a 323 K source. A practical device must therefore connect more than 10^3^ junctions in series. It must also use a substrate that sustains a larger in-plane temperature difference per junction. The bonded route admits the first requirement, because a printed line width rather than a diffusion length defines its junction. The substrate governs the second. Table 3 places these figures beside printed nanotube generators, which deliver microwatts under an applied temperature difference of 60 to 300 K. The applied temperature difference and the number of junctions account for that difference. These two quantities mark the directions in which a cold-source-free device has to develop.

## Figures and Tables

**Table 2:** Substrate properties and steady-state device performance, including the internal resistance and the maximum power.

Substrate	*κ* (W/(m·K)) ^a^	IR Transmittance (%) ^b^	Junction Type	*V*_device_ (mV)	*V*_junction_ (mV)	*R*_int_ (Ω) ^c^	*P*_max_ (nW) ^c^	Δ*T*_eff_ (K)	Temp. Rise (K)	Center − End (K)
PI	0.12	9	Co-filtered	0.04	0.020	50	0.008	0.26	28.1	+0.90
PI	0.12	9	Carbon-paste	0.09	0.045	68	0.030	0.46	24.0	−1.20
COP	0.20	61	Co-filtered	0.30	0.150	48	0.47	1.97	23.6	+1.25
COP	0.20	61	Carbon-paste	0.42	0.210	68	0.65	2.14	25.3	+0.70

^a^ Reference values, not measurements of the present specimens: PI and COP are nominal values for the commercial films used here [44]. Substrate thicknesses were 125 µm (PI) and 100 µm (COP), giving areal thermal resistances of 1.0 × 10^−3^ and 5.0 × 10^−4^ m^2^·K/W, respectively. *V*_device_ is the voltage across the two series-connected junctions; *V*_junction_ = *V*_device_/2. “Temp. rise” is the steady-state film-surface temperature of the center zone minus its own pre-heating value. The center zone contains the junction. “Center − end” is that steady-state center-zone temperature minus the mean of the two end-zone temperatures. ^b^ Blackbody-weighted mean transmittance at 323 K over 2.5–25 µm, computed from the spectra of Figure S4, which are plotted as absorbance *A* with *T* = 10^−^*^A^*. ^c^ *R*_int_ is the internal resistance of the device, that is, of the two series-connected junctions. *P*_max_ = *V*_device_^2^/(4*R*_int_) is the power delivered to a matched load. *R*_int_ refers to the SWCNT material and excludes the contact and interconnect resistances.

**Table 3:** Steady-state performance of the present devices and of reported carbon nanotube thermoelectric generators.

Material and Junction Formation	Junctions	Active Area (cm^2^)	Cold Source	Δ*T* (K) ^b^	V	*R*_int_ (Ω) ^a^	*P*_max_	*P*_max_/(*N*Δ*T*^2^) (nW/K^2^) ^c^	Ref.
SWCNT, co-filtered	2	8	Not used	2.0	0.30 mV	48	0.47 nW	0.060	This work
SWCNT, carbon-paste-bonded	2	8	Not used	2.1	0.42 mV	68	0.65 nW	0.071	This work
SWCNT, co-filtered	2	8	Not used	—	0.38 mV	—	—	—	[44]
SWCNT, mask-assisted printed legs	—	—	Required	300	1.11 V	671	459 µW	—	[59]
SWCNT, screen-printed origami	72	—	Required	60	313 mV	937	25.7 µW	0.10	[60]
SWCNT/poly(aniline-co-acrylonitrile)	—	—	Required	30	—	—	117 nW	—	[61]
Zn-doped Sb_2_Te_3_ film	—	—	Required	40	53.0 mV	—	1100 nW	—	[62]

^a^ The internal resistance of [59] is the reported value, and that of [60] follows from the reported open-circuit voltage and short-circuit current. ^b^ The present devices operate with no cold source. For them, Δ*T* is the effective in-plane temperature difference per junction, Δ*T*_eff_ = *V*/Δ*S*. For the cited devices, Δ*T* is the temperature difference applied across the device. Voltages and powers refer to the complete device in every row. ^c^ *P*_max_/(*N*Δ*T*^2^) = Δ*S*^2^/(4*R*_1_) is the maximum power per junction normalized by the square of the temperature difference, with *R*_1_ the resistance of one junction. The entry is given for the devices that contain *p*–*n* pairs and report the internal resistance. For ref. [60], *N* is the number of *p*–*n* pairs formed by its 143 thermoelements.

## Data Availability

The original contributions presented in this study are included in the article/Supplementary Materials. Further inquiries can be directed to the corresponding author.

## Supplementary Materials

The following supporting information can be downloaded at: https://www.mdpi.com/article/10.3390/ma19194148/s1, Figure S1. Tensile properties of the *p*-type single, *n*-type single, and co-filtered *p*–*n* junction films: (a) tensile strength *σ*, (b) breaking strain *ε*, (c) stress–strain curves, and (d) Young’s modulus *E*. Specimens were scaled to one-tenth of the No. 13B geometry of JIS Z 2241, as in our previous work on SWCNT films [49], and tested with an MX-1000N-FA testing machine (IMADA). This standard is the Japanese equivalent of ISO 6892-1. The parallel portion measured 1.25 mm in width and 5.0 mm in gauge length. Error bars denote one standard deviation over five specimens (*n* = 5). The carbon-paste-bonded film could not be cut to the specified geometry without damaging the joint and was therefore not tensile-tested. In all panels, *p*, *n*, and *p*–*n* on the horizontal axis denote the *p*-type single, *n*-type single, and co-filtered *p*–*n* junction films, respectively; Figure S2. Residuals of the hyperbolic-tangent fits of the Seebeck coefficient profiles of Figure 3a of the main text, plotted against position with the junction at the origin. The residual is the measured Seebeck coefficient minus the value of the hyperbolic-tangent fit, obtained by applying the parameters of Table 1 of the main text to the measured values. The root-mean-square residual is 2.8 µV/K for the co-filtered film and 1.9 µV/K for the carbon-paste-bonded film, that is, 3.7% and 2.0% of the respective total Seebeck coefficient Δ*S*. The residuals show no systematic trend across the transition. Their magnitude reflects the position-to-position variation of the film rather than a departure from the hyperbolic-tangent form. Vertical error bars denote ±1.9 µV/K, and horizontal error bars denote the 5-mm thermocouple separation, as in Figure 3a; Figure S3. (a) Thermal conductivities of the *p*-type and *n*-type SWCNT films and the PI and COP substrates. The SWCNT values are in-plane values measured in our previous substrate study [44] for films prepared by the same route, and the substrate values are nominal, direction-independent figures for the commercial films used here. Areal thermal resistances computed from the substrate values and thicknesses are given in the footnote to Table 2 of the main text. (b) Sheet thermal conductances (thermal conductivity × thickness) of the SWCNT films and the two substrates; the SWCNT columns are drawn at the geometric-mean film thickness and their error bars span the 25–70 µm film-thickness range; Figure S4. Infrared absorbance spectra of the *p*-type single and *n*-type single SWCNT films and the two substrates (PI and COP) over 2.5–25 µm (400–4000 cm^−1^). The vertical dashed line marks the 8.97 µm emission peak of a 323 K blackbody, corresponding to the source temperature of the device measurements. The absorbance *A* is related to the transmittance by *T* = 10^−^*^A^*. The absorbance of the SWCNT films remains at or above approximately 2.0 throughout the range, so that about 1% or less of the incident radiation is transmitted and radiation reaching the film is absorbed in it. PI absorbs strongly at the emission peak, where *A* = 2.4–3.6 over 8.5–9.5 µm, whereas COP has *A* = 0.25 there and remains below about 0.3 over most of the range, interrupted only by narrow absorption bands. Weighting the corresponding transmittance by the 323 K blackbody spectrum gives the effective transmittances quoted in Table 2 of the main text (PI 9%, COP 61%), and their ordering agrees with that of the single-wavelength transmittances at 8.8 µm in our previous report [44]; Figure S5. Transient output voltages of the four devices (two substrates × two junction types) recorded from 5 min before to 10 min after heater activation; the heater is switched on at *t* = 0. Each device contains two series-connected junctions, and the voltages are those measured across the device. The steady-state values quoted in Figure 5a and Table 2 of the main text are the averages over the last minute of the heating period. All four devices pass through a negative excursion within the first two minutes of heating before the output settles at a positive steady-state value. The excursion is deepest for the co-filtered device on PI (−0.58 mV) and shallowest for the carbon-paste-bonded device on COP (−0.26 mV), the remaining two devices lying between them at about −0.3 mV. The excursion arises from the transient polarity mechanism governed by the infrared absorptance and thermal inertia of the substrate, which was reported and modeled separately [44].

## References

[1] Atzori L., Iera A., Morabito G. (2010). The Internet of Things: A survey. Comput. Netw..

[2] Gubbi J., Buyya R., Marusic S., Palaniswami M. (2013). Internet of Things (IoT): A vision, architectural elements, and future directions. Future Gener. Comput. Syst..

[3] Adu-Manu K.S., Adam N., Tapparello C., Ayatollahi H., Heinzelman W. (2018). Energy-harvesting wireless sensor networks (EH-WSNs): A review. ACM Trans. Sens. Netw..

[4] Shaikh F.K., Zeadally S. (2016). Energy harvesting in wireless sensor networks: A comprehensive review. Renew. Sustain. Energy Rev..

[5] Bell L.E. (2008). Cooling, heating, generating power, and recovering waste heat with thermoelectric systems. Science.

[6] Snyder G.J., Toberer E.S. (2008). Complex thermoelectric materials. Nat. Mater..

[7] He J., Tritt T.M. (2017). Advances in thermoelectric materials research: Looking back and moving forward. Science.

[8] Champier D. (2017). Thermoelectric generators: A review of applications. Energy Convers. Manag..

[9] Zebarjadi M., Esfarjani K., Dresselhaus M.S., Ren Z.F., Chen G. (2012). Perspectives on thermoelectrics: From fundamentals to device applications. Energy Environ. Sci..

[10] Vullers R.J.M., van Schaijk R., Doms I., Van Hoof C., Mertens R. (2009). Micropower energy harvesting. Solid-State Electron..

[11] Kim Y.J., Gu H.M., Kim C.S., Choi H., Lee G., Kim S., Yi K.K., Lee S.G., Cho B.J. (2018). High-performance self-powered wireless sensor node driven by a flexible thermoelectric generator. Energy.

[12] Guan M., Wang K., Xu D., Liao W.-H. (2017). Design and experimental investigation of a low-voltage thermoelectric energy harvesting system for wireless sensor nodes. Energy Convers. Manag..

[13] Suarez F., Nozariasbmarz A., Vashaee D., Ozturk M.C. (2016). Designing thermoelectric generators for self-powered wearable electronics. Energy Environ. Sci..

[14] Lee B., Cho H., Park K.T., Kim J.-S., Park M., Kim H., Hong Y., Chung S. (2020). High-performance compliant thermoelectric generators with magnetically self-assembled soft heat conductors for self-powered wearable electronics. Nat. Commun..

[15] Iijima S. (1991). Helical microtubules of graphitic carbon. Nature.

[16] Iijima S., Ichihashi T. (1993). Single-shell carbon nanotubes of 1-nm diameter. Nature.

[17] Bethune D.S., Kiang C.H., de Vries M.S., Gorman G., Savoy R., Vazquez J., Beyers R. (1993). Cobalt-catalysed growth of carbon nanotubes with single-atomic-layer walls. Nature.

[18] Cao Q., Rogers J.A. (2009). Ultrathin films of single-walled carbon nanotubes for electronics and sensors: A review of fundamental and applied aspects. Adv. Mater..

[19] Hu L., Hecht D.S., Gruner G. (2010). Carbon nanotube thin films: Fabrication, properties, and applications. Chem. Rev..

[20] Blackburn J.L., Ferguson A.J., Cho C., Grunlan J.C. (2018). Carbon-nanotube-based thermoelectric materials and devices. Adv. Mater..

[21] MacLeod B.A., Stanton N.J., Gould I.E., Wesenberg D., Ihly R., Owczarczyk Z.R., Hurst K.E., Fewox C.S., Folmar C.N., Holman Hughes K. (2017). Large *n*- and *p*-type thermoelectric power factors from doped semiconducting single-walled carbon nanotube thin films. Energy Environ. Sci..

[22] Komatsu N., Ichinose Y., Dewey O.S., Taylor L.W., Trafford M.A., Yomogida Y., Wehmeyer G., Pasquali M., Yanagi K., Kono J. (2021). Macroscopic weavable fibers of carbon nanotubes with giant thermoelectric power factor. Nat. Commun..

[23] Nakai Y., Honda K., Yanagi K., Kataura H., Kato T., Yamamoto T., Maniwa Y. (2014). Giant Seebeck coefficient in semiconducting single-wall carbon nanotube film. Appl. Phys. Express.

[24] Hata S., Tomotsu J., Gotsubo M., Du Y., Shiraishi Y., Toshima N. (2021). *n*-type carbon nanotube sheets for high in-plane ZT values in double-doped electron-donating graft copolymers containing diphenylhydrazines. Polym. J..

[25] Collins P.G., Bradley K., Ishigami M., Zettl A. (2000). Extreme oxygen sensitivity of electronic properties of carbon nanotubes. Science.

[26] Derycke V., Martel R., Appenzeller J., Avouris P. (2002). Controlling doping and carrier injection in carbon nanotube transistors. Appl. Phys. Lett..

[27] Nonoguchi Y., Ohashi K., Kanazawa R., Ashiba K., Hata K., Nakagawa T., Adachi C., Tanase T., Kawai T. (2013). Systematic conversion of single walled carbon nanotubes into *n*-type thermoelectric materials by molecular dopants. Sci. Rep..

[28] Nonoguchi Y., Nakano M., Murayama T., Hagino H., Hama S., Miyazaki K., Matsubara R., Nakamura M., Kawai T. (2016). Simple salt-coordinated *n*-type nanocarbon materials stable in air. Adv. Funct. Mater..

[29] Nakashima Y., Yamaguchi R., Toshimitsu F., Matsumoto M., Borah A., Staykov A., Islam M.S., Hayami S., Fujigaya T. (2019). Air-stable *n*-type single-walled carbon nanotubes doped with benzimidazole derivatives for thermoelectric conversion and their air-stable mechanism. ACS Appl. Nano Mater..

[30] Horike S., Wei Q., Akaike K., Kirihara K., Mukaida M., Koshiba Y., Ishida K. (2022). Bicyclic-ring base doping induces *n*-type conduction in carbon nanotubes with outstanding thermal stability in air. Nat. Commun..

[31] Amma Y., Miura K., Nagata S., Nishi T., Miyake S., Miyazaki K., Takashiri M. (2022). Ultra-long air-stability of *n*-type carbon nanotube films with low thermal conductivity and all-carbon thermoelectric generators. Sci. Rep..

[32] Yamamoto H., Amezawa T., Okano Y., Hoshino K., Ochiai S., Sunaga K., Miyake S., Takashiri M. (2025). High thermal durability and thermoelectric performance with ultra-low thermal conductivity in *n*-type single-walled carbon nanotube films by controlling dopant concentration with cationic surfactant. Appl. Phys. Lett..

[33] Mizuno K., Ishii J., Kishida H., Hayamizu Y., Yasuda S., Futaba D.N., Yumura M., Hata K. (2009). A black body absorber from vertically aligned single-walled carbon nanotubes. Proc. Natl. Acad. Sci. USA.

[34] St-Antoine B.C., Ménard D., Martel R. (2009). Position sensitive photothermoelectric effect in suspended single-walled carbon nanotube films. Nano Lett..

[35] DeBorde T., Aspitarte L., Sharf T., Kevek J.W., Minot E.D. (2014). Photothermoelectric effect in suspended semiconducting carbon nanotubes. ACS Nano.

[36] Li K., Kinoshita Y., Sakai D., Kawano Y. (2023). Recent progress in development of carbon-nanotube-based photo-thermoelectric sensors and their applications in ubiquitous non-destructive inspections. Micromachines.

[37] Wang H., Wang R., Chen C., Zhou Z., Liu J.-W. (2022). Manipulating single-walled carbon nanotube arrays for flexible photothermoelectric devices. JACS Au.

[38] Komori T., Tamai R., Nakazawa Y., Hoshino K., Abe H., Tanaka S., Takashiri M. (2024). Stable photothermal conversion in single-walled carbon nanotube device with *pn*-junction under uniform sunlight irradiation. Mater. Today Commun..

[39] Norimasa O., Tamai R., Nakayama H., Shinozaki Y., Takashiri M. (2025). Self-generated temperature gradient under uniform heating in *p*–*i*–*n* junction carbon nanotube thermoelectric generators. Sci. Rep..

[40] Chiba T., Amma Y., Takashiri M. (2021). Heat source free water floating carbon nanotube thermoelectric generators. Sci. Rep..

[41] Komori T., Norimasa O., Yamamoto H., Hoshino K., Takada Y., Takashiri M. (2023). Effect of Seebeck coefficient distribution across *pn*-junction in carbon nanotube films for photothermoelectric power generation by localized sunlight irradiation. Diam. Relat. Mater..

[42] Yamaguchi R., Ishii T., Matsumoto M., Borah A., Tanaka N., Oda K., Tomita M., Watanabe T., Fujigaya T. (2021). Thermal deposition method for *p*–*n* patterning of carbon nanotube sheets for planar-type thermoelectric generator. J. Mater. Chem. A.

[43] Moore A.L., Shi L. (2014). Emerging challenges and materials for thermal management of electronics. Mater. Today.

[44] Tamai R., Nakayama H., Ochiai S., Takashiri M. (2026). Substrate engineering of SWCNT *p*–*n* junctions for dual-mode power generation and heat-flux sensing. Adv. Sci..

[45] Zhang Z.M., Lefever-Button G., Powell F.R. (1998). Infrared refractive index and extinction coefficient of polyimide films. Int. J. Thermophys..

[46] Kamyshny A., Magdassi S. (2014). Conductive nanomaterials for printed electronics. Small.

[47] Mytafides C.K., Tzounis L., Karalis G., Formanek P., Paipetis A.S. (2021). High-power all-carbon fully printed and wearable SWCNT-based organic thermoelectric generator. ACS Appl. Mater. Interfaces.

[48] Seki Y., Takashiri M. (2020). Freestanding bilayers of drop-cast single-walled carbon nanotubes and electropolymerized poly(3,4-ethylenedioxythiophene) for thermoelectric energy harvesting. Org. Electron..

[49] Yamamoto H., Okano Y., Uchida K., Kageshima M., Kuzumaki T., Miyake S., Takashiri M. (2024). Phonon transports in single-walled carbon nanotube films with different structures determined by tensile tests and thermal conductivity measurements. Carbon Trends.

[50] Smits F.M. (1958). Measurement of sheet resistivities with the four-point probe. Bell Syst. Tech. J..

[51] Ziolkowski P., Wambach M., Ludwig A., Mueller E. (2018). Application of high-throughput Seebeck microprobe measurements on thermoelectric half-Heusler thin film combinatorial material libraries. ACS Comb. Sci..

[52] Kumanek B., Janas D. (2019). Thermal conductivity of carbon nanotube networks: A review. J. Mater. Sci..

[53] Han Z., Fina A. (2011). Thermal conductivity of carbon nanotubes and their polymer nanocomposites: A review. Prog. Polym. Sci..

[54] Mallick M.M., Franke L., Hussein M., Rösch A.G., Long Z., Eggeler Y.M., Lemmer U. (2024). Printed lateral *p*–*n* junction for thermoelectric generation. Small Sci..

[55] Tanaka N., Fujigaya T. (2026). *p*–*n* patterning strategies for single-walled carbon nanotubes for the fabrication of joint-free thermoelectric generators. Polym. J..

[56] Dai X., Wang Y., Li K., Li G., Wang J., Sun X., Zhang L., Wang H. (2021). Joint-free single-piece flexible thermoelectric devices with ultrahigh resolution *p*–*n* patterns toward energy harvesting and solid-state cooling. ACS Energy Lett..

[57] Tanaka N., Yamamoto M., Yamaguchi I., Hamasuna A., Honjo E., Fujigaya T. (2023). Photolithographic *p*–*n* patterning of single-walled carbon nanotube sheets using photobase generators. J. Mater. Chem. A.

[58] Suzuki D., Terasaki N. (2024). Aerosol doping system for microscale seamless *p*–*n* patterning of carbon nanotube films. ACS Appl. Mater. Interfaces.

[59] Mytafides C.K., Tzounis L., Karalis G., Formanek P., Paipetis A.S. (2021). Fully printed and flexible carbon nanotube-based thermoelectric generator capable for high-temperature applications. J. Power Sources.

[60] Simopoulos F., Karalis G., Krassas M., Porfyrakis E., Kampourakis G., Katsiaounis S., Papagelis K., Formanek P., Kavvadias I.E., Papadakis N.C. (2026). Screen-printed origami-inspired paper-based carbon nanotube thermoelectric generators with ultra-high power output. ACS Appl. Electron. Mater..

[61] Erden F., Danaci I., Ozbay S. (2025). Flexible thermoelectric generators based on single-walled carbon nanotube/poly(aniline-*co*-acrylonitrile) composites. Adv. Electron. Mater..

[62] Zhang Z.-L., Yang W.-Y., Wu B., Nisar M., Li F., Liang G.-X., Luo J.-T., Chen Y.-X., Zheng Z.-H. (2025). A Zn-doped Sb_2_Te_3_ flexible thin film with decoupled Seebeck coefficient and electrical conductivity via band engineering. Chem. Sci..

