# Supplementary material for: Co-Filtered Versus Carbon-Paste-Bonded p–n Junctions in Cold-Source-Free Single-Walled Carbon Nanotube Thermoelectric Generators
Source: Materials (Basel). 2026 Sep 29;19(19):4148. doi: 10.3390/ma19194148 (PMC13666075; doi:10.3390/ma19194148)
Supplement: Supplementary file 1 [file materials-19-04148-s001.zip › materials-4541911-Supplementary.pdf]

Supplementary Materials

Co-Filtered versus Carbon-Paste-Bonded *p–n* Junctions in Cold-Source-Free Single-Walled Carbon Nanotube Thermoelectric Generators

Hiroto Nakayama, Ryota Tamai, Yuta Asano, and Masayuki Takashiri

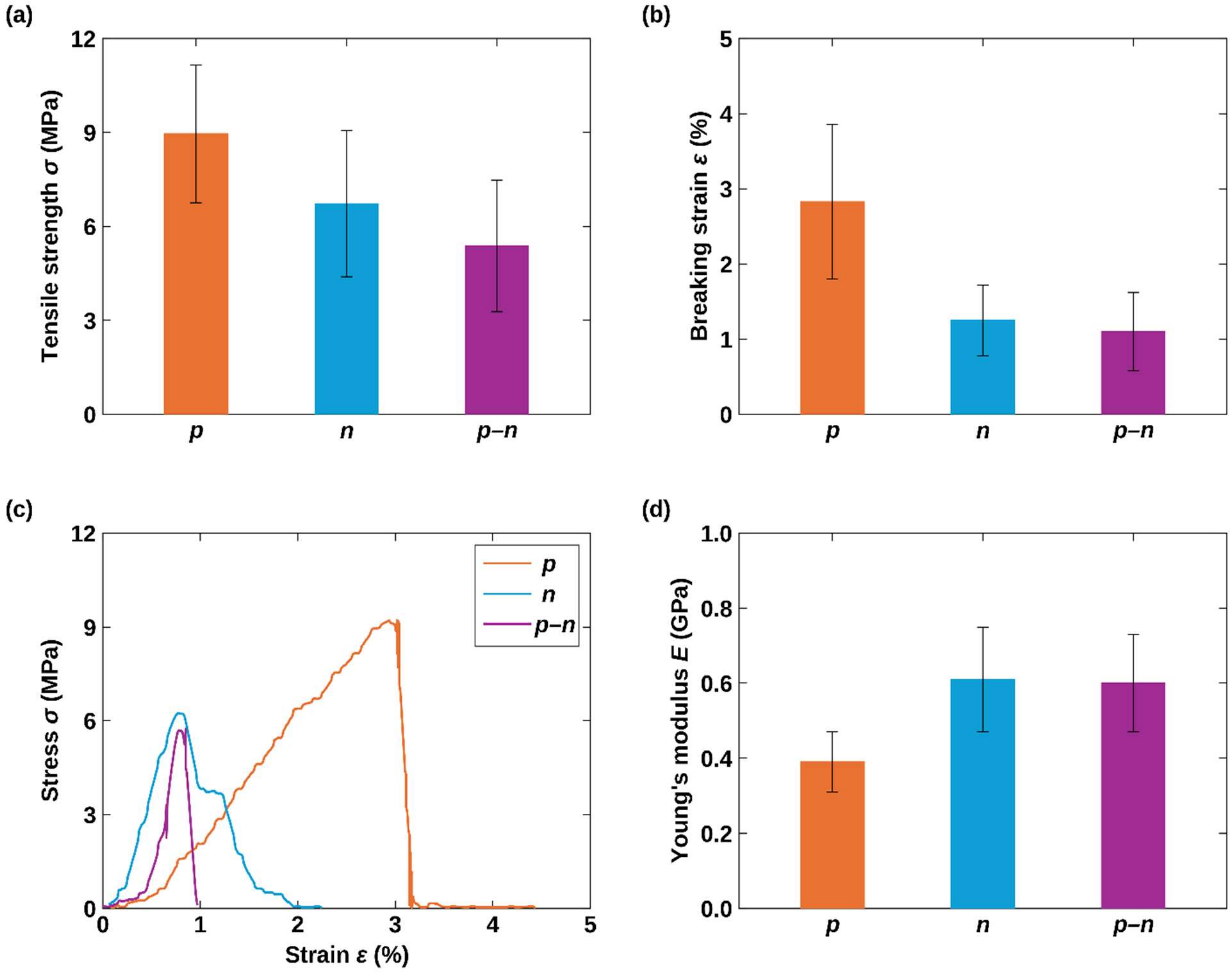

Figure S1. Tensile properties of the *p*-type single, *n*-type single, and co-filtered *p–n* junction films: (a) tensile strength σ, (b) breaking strain ε, (c) stress–strain curves, and (d) Young's modulus E. Specimens were scaled to one-tenth of the No. 13B geometry of JIS Z 2241, as in our previous work on SWCNT films [S1], and tested with an MX-1000N-FA testing machine (IMADA). This standard is the Japanese equivalent of ISO 6892-1. The parallel portion measured 1.25 mm in width and 5.0 mm in gauge length. Error bars denote one standard deviation over five specimens (n = 5). The carbon-paste-bonded film could not be cut to the specified geometry without damaging the joint and was therefore not tensile-tested. In all panels, *p*, *n*, and *p–n* on the horizontal axis denote the *p*-type single, *n*-type single, and co-filtered *p–n* junction films, respectively.

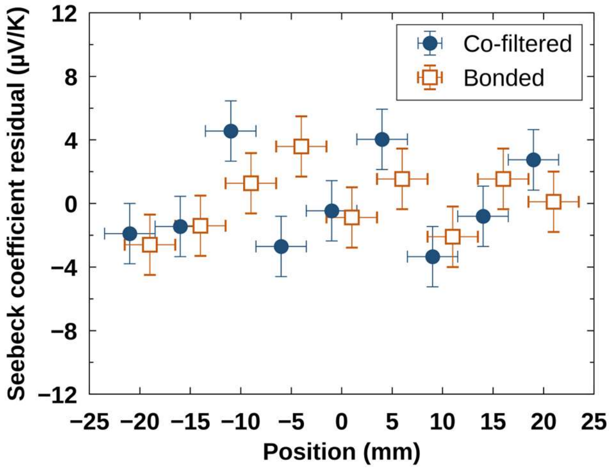

Figure S2. Residuals of the hyperbolic-tangent fits of the Seebeck coefficient profiles of Figure 3a of the main text, plotted against position with the junction at the origin. The residual is the measured Seebeck coefficient minus the value of the hyperbolic-tangent fit, obtained by applying the parameters of Table 1 of the main text to the measured values. The root-mean-square residual is 2.8 μV/K for the co-filtered film and 1.9 μV/K for the carbon-paste-bonded film, that is, 3.7% and 2.0% of the respective total Seebeck coefficient ΔS. The residuals show no systematic trend across the transition. Their magnitude reflects the position-to-position variation of the film rather than a departure from the hyperbolic-tangent form. Vertical error bars denote ±1.9 μV/K, and horizontal error bars denote the 5-mm thermocouple separation, as in Figure 3a.

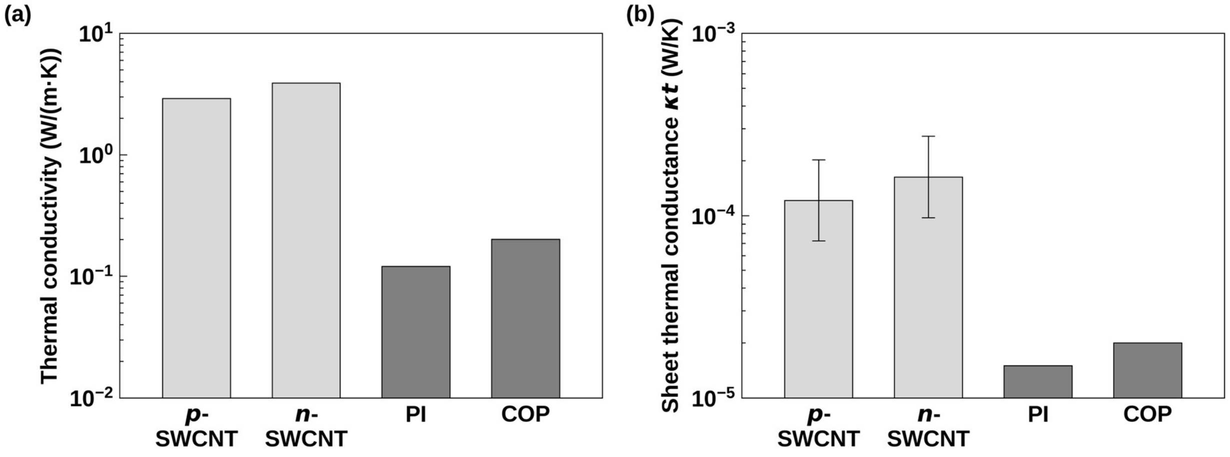

Figure S3. (a) Thermal conductivities of the *p*-type and *n*-type SWCNT films and the PI and COP substrates. The SWCNT values are in-plane values measured in our previous substrate study [S2] for films prepared by the same route, and the substrate values are nominal, direction-independent figures for the commercial films used here. Areal thermal resistances computed from the substrate values and thicknesses are given in the footnote to Table 2 of the main text. (b) Sheet thermal conductances (thermal conductivity × thickness) of the SWCNT films and the two substrates; the SWCNT columns are drawn at the geometric-mean film thickness and their error bars span the 25–70 μm film-thickness range.

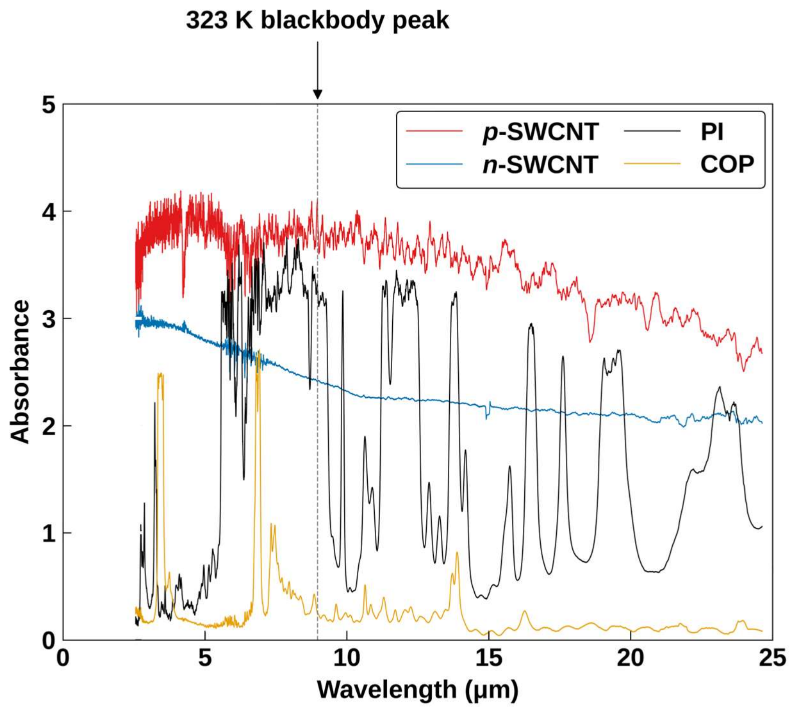

Figure S4. Infrared absorbance spectra of the *p*-type single and *n*-type single SWCNT films and the two substrates (PI and COP) over 2.5–25 μm (400–4000 cm^{-1}). The vertical dashed line marks the 8.97 μm emission peak of a 323 K blackbody, corresponding to the source temperature of the device measurements. The absorbance A is related to the transmittance by $T = 10^{-A}$. The absorbance of the SWCNT films remains at or above approximately 2.0 throughout the range, so that about 1% or less of the incident radiation is transmitted and radiation reaching the film is absorbed in it. PI absorbs strongly at the emission peak, where A = 2.4–3.6 over 8.5–9.5 μm, whereas COP has A = 0.25 there and remains below about 0.3 over most of the range, interrupted only by narrow absorption bands. Weighting the corresponding transmittance by the 323 K blackbody spectrum gives the effective transmittances quoted in Table 2 of the main text (PI 9%, COP 61%), and their ordering agrees with that of the single-wavelength transmittances at 8.8 μm in our previous report [S2].

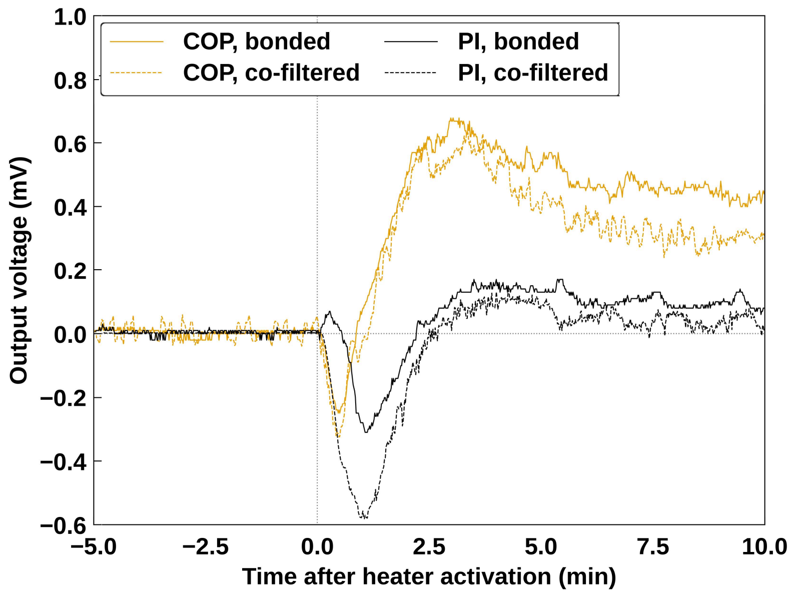

Figure S5. Transient output voltages of the four devices (two substrates × two junction types) recorded from 5 min before to 10 min after heater activation; the heater is switched on at $t = 0$. Each device contains two series-connected junctions, and the voltages are those measured across the device. The steady-state values quoted in Figure 5a and Table 2 of the main text are the averages over the last minute of the heating period. All four devices pass through a negative excursion within the first two minutes of heating before the output settles at a positive steady-state value. The excursion

is deepest for the co-filtered device on PI (−0.58 mV) and shallowest for the carbon-paste-bonded device on COP (−0.26 mV), the remaining two devices lying between them at about −0.3 mV. The excursion arises from the transient polarity mechanism governed by the infrared absorptance and thermal inertia of the substrate, which was reported and modeled separately [S2].

References

S1. Yamamoto, H.; Okano, Y.; Uchida, K.; Kageshima, M.; Kuzumaki, T.; Miyake, S.; Takashiri, M. Phonon transports in single-walled carbon nanotube films with different structures determined by tensile tests and thermal conductivity measurements. *Carbon Trends* **2024**, *17*, 100435. https://doi.org/10.1016/j.cartre.2024.100435

S2. Tamai, R.; Nakayama, H.; Ochiai, S.; Takashiri, M. Substrate engineering of SWCNT *p–n* junctions for dual-mode power generation and heat-flux sensing. *Adv. Sci.* **2026**, e76889. https://doi.org/10.1002/advs.76889
